# Prognostic significance of neutrophil-to-lymphocyte ratio and platelet-to-lymphocyte ratio in non-small cell lung cancer

**DOI:** 10.1097/MD.0000000000034180

**Published:** 2023-06-30

**Authors:** Mesut Bayraktaroglu, Birsen Pinar Yildiz

**Affiliations:** a Istanbul Bahcelievler Memorial Hospital, Istanbul, Turkey; b Yedikule Thoracic Disease and Surgery Training and Research Hospital, Istanbul, Turkey.

**Keywords:** malignancy, prognostic factors, survival

## Abstract

Non-small cell lung cancer (NSCLC) is characterized by diagnosis at an advanced stage, low rate of operability and poor survival. Therefore, there is a need for a biomarker in NSCLC patients to predict the likely outcome and to accurately stratify the patients in terms of the most appropriate treatment modality. To evaluate prognostic value of pretreatment neutrophil-to-lymphocyte ratio (NLR) and platelet-to-lymphocyte ratio (PLR) in NSCLC. A total of 124 NSCLC patients (mean ± standard deviation age: 60.7 ± 9.3 years, 94.4% were males) were included in this retrospective study. Data were retrieved from the hospital records. The association of NLR and PLR with clinicopathological factors and overall survival was analyzed. One-year, 2-year and 5-year survival rates were 59.2%, 32.0%, and 16.2%, respectively. Median duration of survival was shorter in patient groups with elevated NLR and PLR. Five-year survival rate was quite lower in patient groups with elevated NLR and PLR. Hazard rate (HR) for mortality was 1.76 (95% confidence interval [CI]: 1.19–2.61, *P* = .005) for NLR ≥ 3 over NLR < 3. HR was 1.64 (95%CI: 1.11–2.42, *P* = .013) for PLR ≥ 150 over PLR < 150. Cox-regression analysis revealed that, when adjusted for other independent predictors of survival, NLR and PLR still remain significant predictors of poorer survival. Our findings indicate that elevated pretreatment NLR and PLR are associated with advanced disease and poor survival in NSCLC patients, NLR and PLR values are correlated with each other.

## 1. Introduction

Non-small cell lung cancer (NSCLC), accounting for >85% of lung cancers, is characterized by diagnosis at an advanced stage, low rate of operability and poor survival despite efforts and progress in the diagnosis and treatment.^[1–3]^ Therefore, there is a need for reliable and inexpensive prognostic biomarker in NSCLC patients to predict the likely outcome and to accurately stratify the patients in terms of the most appropriate treatment modality.^[4,5]^

It is widely acknowledged that systemic inflammatory response serves a critical role in the pathogenesis and progression of cancer.^[6,7]^ Being considered as convenient, reliable and inexpensive biomarkers of systemic inflammation, neutrophil-to-lymphocyte (NLR) and the platelet-to-lymphocyte ratio (PLR) are known to be associated with the tumor progression and survival outcome in different types of cancer including the lung cancer.^[8–13]^

Although some studies have indicated the prognostic role of NLR and PLR in NSCLC patients,^[8,14,15]^ others reported no association of PLR with survival^[16,17]^ and there is controversy regarding the utility of these markers in all subsets of NSCLC patients, particularly in early-stage NSCLC as well as limited data on the association of NLR and PLR with clinicopathological factors.^[4,5]^

This study was therefore designed to evaluate prognostic significance of pretreatment NLR and PLR in terms of clinicopathological factors and survival outcome in NSCLC patients with early-stage or locally advanced disease.

## 2. Materials and methods

### 2.1. Study population

A total of 124 treatment-naïve patients (mean ± standard deviation age: 60.7 ± 9.3 years, 94.4% were males) newly diagnosed with NSCLC in 2010 to 2011 were included in this study. We have designed and started collecting data in 2017 in this retrospective cohort study. Median follow-up duration was 14.6 months (0.5–78). Presence of NSCLC histology and availability of data on complete blood count (CBC) analysis including leukocyte subtypes in the pretreatment period were the inclusion criteria of the study. Patients with co-morbid hematologic disorders or autoimmune diseases were excluded from the study.

The study was conducted in accordance with the ethical principles stated in the “Declaration of Helsinki” and approved by the Research Ethics Committee of Istanbul University Istanbul Faculty of Medicine, including permission for the use of patient data for publication purposes (Date of Approval: November 10, 2010. Protocol No: 849). Informed consent was not necessary in this case as patients were diagnosed and treated in accordance with established global guidelines. The testing and recording of CBC for each patient were conducted as routine care procedures, without any additional research-related intentions.

### 2.2. Assessments

Data on patient demographics, preoperative diagnosis and staging and pretreatment CBC results including neutrophil, lymphocyte, and platelet counts were retrieved from the hospital records. The neutrophil-to-lymphocyte ratio (NLR) and PLR were calculated as the neutrophil and platelet counts divided by the lymphocyte count, respectively. The histopathological findings were classified according to the World Health Organization guidelines, and the pathological disease stages were described according to the 7th tumor-node-metastasis (TNM) staging system for NSCLC.^[18]^

### 2.3. Study parameters

Analyses were performed for the whole patient population and also in subgroups defined by demographic parameters, clinicopathological characteristics, hematologic parameters, NLR and PLR.

For some analyses, patients were classified according to NLR and PLR values. The following explanations are for NLR, but they are valid for PLR as they are, as well. The quartiles are equal quarter parts of patient population, that is, first quartile (Q1) is constituted by patients whose NLR value is lower than 25^th^ percentile, second quartile (Q2) is constituted by patients whose NLR value is between 25^th^ and 50^th^ percentiles, third quartile (Q3) is constituted by patients whose NLR value is between 50^th^ and 75^th^ percentiles, and fourth quartile (Q4) is constituted by patients whose NLR value is higher than 75^th^ percentile. With that approach, it is ensured that the patients are divided into 4 equal subgroups. In addition to being classified by quartiles, the patients were also divided into 2 equal subgroups, by using cutoffs (rounded values close to median and compatible with the literature), ensuring that the patients were divided into 2 equal subgroups. These cutoff values were determined as ≥3 and ≥150 for NLR and PLR, respectively.

### 2.4. Statistical analysis

Statistical analysis was made using International Business Machines statistical package for the social sciences Statistics for Windows, version 22.0 (International Business Machines Corp., Armonk, NY). Chi-square (*χ*^2^) test and Mantel-Haenszel test were used for the comparison of categorical data, while numerical data were analyzed by means of Student *t* test and One-way analysis of variance (with post hoc Tukey test) for variables with normal distribution and by means of Mann-Whitney U and Kruskal Wallis tests for non-normally distributed variables. Survival analysis was made by Kaplan–Meier analysis and comparisons were made by log-rank test. Factors associated with survival were initially evaluated by univariate Cox regression analysis. Then, multivariate Cox regression analysis was performed entering some variables as predictors. These variables which were chosen as predictors were the ones which significantly differ among NLR and PLR quartiles, and other variables which have been shown to have independent significant association with survival in the univariate analysis (by Kaplan–Meier survival analysis or Cox regression analysis). Accordingly, the predictive role of NLR and PLR in survival as well as the factors with significant impact on their association with survival was analyzed by multivariate analysis adjusted for independent predictors of survival. Cox regression models were run twice; first by entering all selected predictors into the model, regardless of their individual p values, and second by entering only significant predictors (*P* value < .10) which were selected with backward stepwise elimination approach. In this backward stepwise elimination, all variables are first entered into the model, then the most insignificant variable is eliminated, and the model is run second time with the remaining variables. The most insignificant variable in this second model is then eliminated and the model is run third time with the remaining variables. These cycles are repeated until all the remaining variables are significant (*P* value < .10). Hazard ratios (HRs) were estimated using the Cox regression analysis and were reported with their corresponding 95% confidence intervals (CIs). Data were expressed as mean ± standard deviation, median (minimum-maximum), 95%CI and percent (%) where appropriate. *P* < .05 was considered statistically significant.

## 3. Results

Median age of patients was 61.5 years (34–80 years) and males composed the vast majority (n = 117; 94.4%) of study population. Squamous cell carcinoma (41.1%) was the most commonly noted NSCLC subtype, while TNM stage IV disease was evident in 42.3% of patients and 82.3% of patients were inoperable at the time of diagnosis.

### 3.1. NLR and PLR

Mean NLR was 3.85 ± 3.21 (0.94–26.6). Median NLR was 2.92; accordingly patients were divided half and half to 2 groups with NLR ≥ 3 and NLR < 3. Mean PLR was 188.2 ± 99.2 (70.9–708.5). Median PLR was 168.6. Patients were divided half and half to 2 groups with PLR ≥ 150 and PLR < 150.

### 3.2. The relationship between NLR and other prognostic variables

It was found that NLR quartiles were associated with tumor stage and operability. The patients in higher NLR quartiles had more advanced stage (*P* = .030). Higher NLR quartiles had lower chance of operability (38.7%, 12.9%, 12.9%, and 6.5% in Q1, Q2, Q3, and Q4, respectively, *P* = .002).

Some hematologic parameters including white blood cell (WBC), platelet count, plateletcrit, neutrophil count and percent and monocyte count, showed positive correlation with NLR, that is, they increased in values with increasing NLR values, when moving from Q1 to Q4. PLR also showed parallel increases in values with increasing NLR values, when moving from Q1 to Q4. The proportion of patients with PLR ≥ 150 showed increase with increasing NLR quartiles (9.7%, 38.7%, 61.3%, and 90.3% in Q1, Q2, Q3, and Q4, respectively, *P* < .001) (Table 1).

**Table 1:** Prognostic factors in NLR and PLR quartiles.

	NLR quartiles				
	Q1 (n = 31)	Q2 (n = 31)	Q3 (n = 31)	Q4 (n = 31)	*P* value
NLR, mean ± SD	1.8 ± 0.3	2.5 ± 0.2	3.6 ± 0.3	7.5 ± 4.7	-
PLR, mean ± SD	118.7 ± 31.1	165.3 ± 68.3	182.7 ± 52.9	286.2 ± 130.4	<.001*
PLR category, n (%)					
<150	28 (90.3)	19 (61.3)	12 (38.7)	3 (9.7)	<.001**
≥150	3 (9.7)	12 (38.7)	19 (61.3)	28 (90.3)	
PLR quartiles, n (%)					
Q1	18 (58.1)	10 (32.3)	2 (6.5)	1 (3.2)	<.001**
Q2	10 (32.3)	9 (29.0)	10 (32.3)	2 (6.5)	
Q3	3 (9.7)	7 (22.6)	14 (45.2)	7 (22.6)	
Q4	0 (0.0)	5 (16.1)	5 (16.1)	21 (66.7)	
	NLR quartiles				
	Q1 (n = 31)	Q2 (n = 31)	Q3 (n = 31)	Q4 (n = 31)	*P* value
PLR, mean ± SD	101.3 ± 14.6	142.6 ± 14.2	186.8 ± 13.2	322.3 ± 108.4	-
NLR, mean ± SD	2.16 ± 0.69	2.80 ± 0.94	3.55 ± 1.17	6.87 ± 5.09	<.001*
NLR category, n (%)					
<3	28 (90.3)	19 (61.3)	10 (32.3)	5 (16.1)	<.001**
≥3	3 (9.7)	12 (38.7)	21 (67.7)	26 (83.9)	
NLR quartiles, n (%)					
Q1	18 (58.1)	10 (32.3)	3 (9.7)	0 (0.0)	<.001**
Q2	10 (32.3)	9 (29.0)	7 (22.6)	5 (16.1)	
Q3	2 (6.5)	10 (32.3)	14 (45.2)	5 (16.1)	
Q4	1 (3.2)	2 (6.5)	7 (22.6)	21 (67.7)	

ANOVA = analysis of variance, NLR = neutrophil-to-lymphocyte ratio, PLR = platelet-to-lymphocyte ratio, Q = quartile, SD = standard deviation.

* One-way ANOVA.

** Mantel-Haenszel test.

On the other hand, some hematologic parameters including red blood cell, hemoglobin, hematocrit, lymphocyte count and percent, showed negative correlation with NLR, that is, they decreased in values with increasing NLR values, when moving from Q1 to Q4.

### 3.3. The relationship between PLR and other prognostic variables

It was found that PLR quartiles were associated with TNM stage; the patients in higher PLR quartiles had more advanced stage (*P* = .043).

Some hematologic parameters including WBC, platelet count, platelecrit, neutrophil count and percent, showed positive correlation with PLR, that is, they increased in values with increasing PLR values, when moving from Q1 to Q4. NLR also showed parallel increases in values with increasing PLR values, when moving from Q1 to Q4. The proportion of patients with NLR ≥ 3 showed increase with increasing PLR quartiles (9.7%, 38.7%, 67.7%, and 83.9% in Q1, Q2, Q3, and Q4, respectively, *P* < .001) (Table 1).

On the other hand, some hematologic parameters including red blood cell, hemoglobin, hematocrit, mean corpuscular volume, mean corpuscular hemoglobin, mean platelet volume, lymphocyte count and percent, showed negative correlation with PLR, that is, they decreased in values with increasing PLR values, when moving from Q1 to Q4.

### 3.4. Survival in patient subgroups

At the end of follow-up period, 104 (83.9%) patients had died and 20 (16.1%) patients were still alive. Overall, 1-year, 2-year, and 5-year survival rates were 59.2%, 32.0%, and 16.2%, respectively. The median duration of survival was 14.8 months (95%CI: 11.3–18.2) (Table 2). The median duration of survival was significantly associated with the tumor stage (*P* < .001). Patients in stage IV had the shortest survival duration, which is 7.3 months (95%CI: 5.4–9.1). Operability was another significant prognostic factor. Median duration of survival was significantly shorter in inoperable than operable patients (12.4 vs 60.4 months, *P* < .001) (Table 2). Age, tumor dimension, WBC, neutrophil count, neutrophil percent and basophil count were determined as bad prognostic factors. Hemoglobin, hematocrit, mean corpuscular hemoglobin, lymphocyte percent were found to be good prognostic factors (Table 3).

**Table 2:** Survival analysis in patient subgroups (Kaplan–Meier analysis).

	Survival (%) (mean ± SE)					Duration of survival (mo)	*P* value
	1-yr	2-yr	3-yr	4-yr	5-yr	Median (95%CI)	
Overall	59.2 ± 4.4	32.0 ± 4.2	21.8 ± 3.8	20.1 ± 3.7	16.2 ± 3.4	14.8 (11.3–18.2)	
Operability							<.001
Inoperable	53.5 ± 5.0	23.8 ± 4.2	12.2 ± 3.3	10.0 ± 3.1	8.5 ± 2.9	12.4 (7.8–16.9)	
Operable	85.7 ± 7.6	71.4 ± 9.9	66.7 ± 10.3	66.7 ± 10.3	51.6 ± 11.1	60.4 (51.6–69.2)	
NLR category							.004
<3	64.2 ± 6.1	44.4 ± 6.4	30.9 ± 6.0	29.1 ± 5.9	23.0 ± 5.6	19.9 (10.8–29.1)	
≥3	54.1 ± 6.4	19.7 ± 5.1	12.9 ± 4.3	11.1 ± 4.1	9.2 ± 3.8	12.3 (6.1–18.6)	
NLR quartiles							<.001
Q1	71.0 ± 8.2	51.6 ± 9.0	38.0 ± 8.8	38.0 ± 8.8	30.7 ± 8.5	24.1 (11.4–36.7)	Ref.
Q2	57.1 ± 9.0	37.0 ± 8.8	23.5 ± 7.8	20.2 ± 7.3	15.1 ± 7.0	12.7 (8.8–16.6)	.126
Q3	56.7 ± 9.0	26.7 ± 8.1	20.0 ± 7.3	16.7 ± 6.8	-	14.3 (3.8–24.8)	.051
Q4	51.6 ± 9.0	12.9 ± 6.0	4.8 ± 4.3	4.8 ± 4.3	0.0 ± 0.0	12.1 (4.4–19.7)	<.001
PLR category							.012
<150	62.9 ± 6.1	38.7 ± 6.2	27.0 ± 5.7	27.0 ± 5.7	23.2 ± 5.5	16.8 (11.7–21.9)	
≥150	55.4 ± 6.4	25.2 ± 5.6	16.5 ± 4.8	12.8 ± 4.4	8.8 ± 3.9	12.4 (6.9–17.8)	
PLR quartiles							.007
Q1	61.3 ± 8.7	41.9 ± 8.9	28.2 ± 8.2	28.2 ± 8.2	24.6 ± 7.9	16.8 (5.4–28.2)	Ref.
Q2	64.5 ± 8.6	35.5 ± 8.6	25.8 ± 7.9	25.8 ± 7.9	22.1 ± 7.6	18.3 (13.2–23.4)	.913
Q3	63.3 ± 8.8	30.0 ± 8.4	22.9 ± 7.8	15.2 ± 6.8	11.4 ± 6.1	14.3 (8.2–20.4)	.189
Q4	47.5 ± 9.1	20.4 ± 7.4	10.2 ± 5.6	10.2 ± 5.6	6.8 ± 4.6	7.7 (0.0–15.4)	.026

CI = confidence interval, NLR = neutrophil-to-lymphocyte ratio, NSCLC = non-small cell lung cancer, PLR = platelet-to-lymphocyte ratio, Ref = reference, SE = standard error.

**Table 3:** Significant prognostic factors for survival (univariate Cox regression analysis).

	HR	95%CI		*P*
NLR	1.083	1.031	1.138	.002
NLR ≥ 3	1.759	1.188	2.607	.005
NLR quartiles				.004
Q1 vs Q2	0.631	0.355	1.123	.118
Q2 vs Q3	0.892	0.515	1.546	.684
Q3 vs Q4	0.630	0.370	1.072	.089
PLR	1.003	1.001	1.005	.001
PLR ≥ 150	1.639	1.108	2.423	.013
PLR quartiles				.042
Q1 vs Q2	0.949	0.535	1.684	.859
Q2 vs Q3	0.733	0.425	1.265	.264
Q3 vs Q4	0.704	0.416	1.191	.191
Age (yr)	1.033	1.011	1.056	.003
Tumor diameter (mm)	1.010	1.003	1.017	.005
Operability	0.249	0.132	0.472	<.001
WBC count (10^9^/L)	1.081	1.020	1.145	.008
Hemoglobin (g/dL)	0.862	0.767	0.969	.013
Hematocrit (%)	0.956	0.917	0.997	.034
MCH (pg)	0.928	0.866	0.996	.037
Neutrophil				
Count (10^9^/L)	1.100	1.037	1.168	.002
Percent	1.035	1.013	1.058	.002
Lymphocyte				
Count (10^9^/L)	0.759	0.561	1.028	.075
Percent	0.954	0.931	0.977	<.001
Basophil				
Count (10^9^/L)	10.421	2.837	38.278	<.001
Percent	1.066	0.974	1.167	.165

CI *=* confidence interval, HR *=* hazard rate, MCH *=* mean corpuscular hemoglobin, NLR *=* neutrophil-to-lymphocyte ratio, NSCLC *=* non-small cell lung cancer, PLR = platelet-to-lymphocyte ratio, RBC *=* red blood cell, WBC *=* White blood cell.

### 3.5. The association between NLR and survival

**Figure 1.**
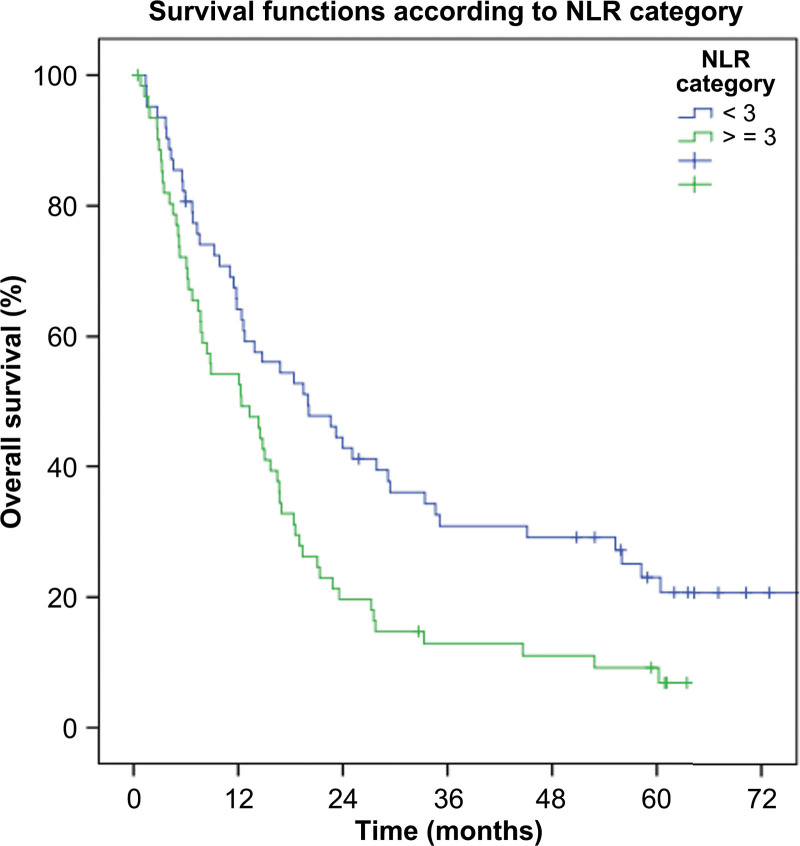
Kaplan–Meier survival curves for NLR categories (<3 vs ≥3). NLR = neutrophil-to-lymphocyte ratio.

NLR was found to be a significant bad prognostic factor. Median duration of survival was significantly longer in patients with NLR < 3 versus NLR ≥ 3 (19.9 vs 12.3 months, *P* = .004) (Table 2 and Fig. 1).

HR of patients whose NLR value was ≥ 3 over patients whose NLR value was < 3 was 1.76 (95%CI: 1.19–2.61) (*P* = .005) (Table 3).

Cox-regression analysis revealed that, when adjusted for other independent predictors of survival (age, tumor stage, pathological type, operability, WBC count, neutrophil count) (Supplementary table 1, http://links.lww.com/MD/J214), NLR still remains a significant predictor of poorer survival (HR: 1.232, 95%CI: 1.081–1.403, *P* = .002).

### 3.6. The association between PLR and survival

**Figure 2.**
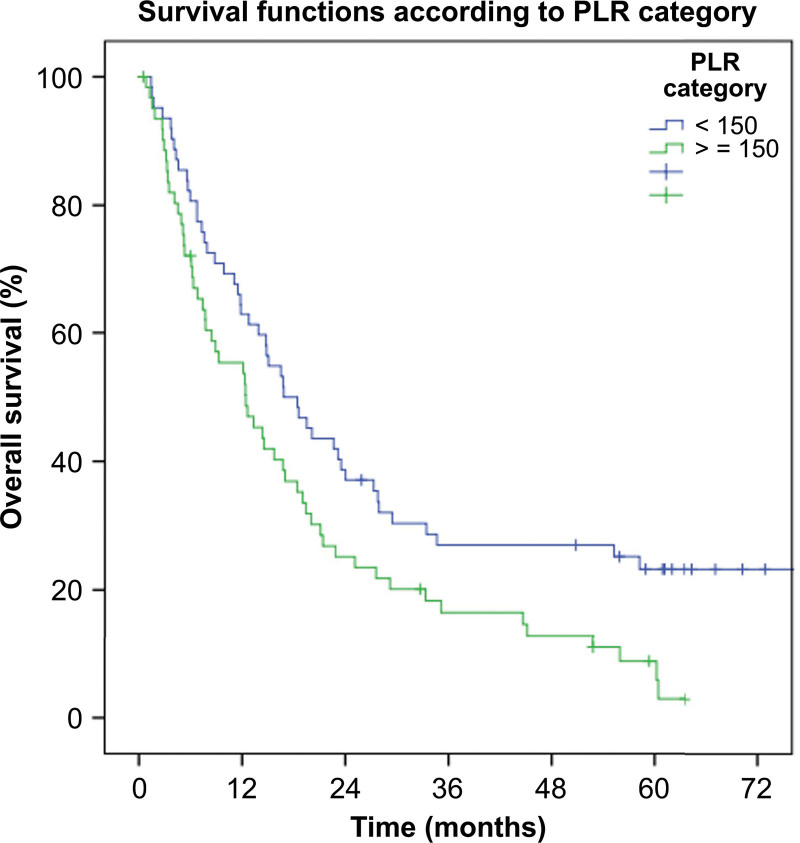
Kaplan–Meier survival curves for PLR categories (<150 vs ≥150). PLR = platelet-to-lymphocyte ratio.

PLR was also another significant bad prognostic factor. Median duration of survival was significantly longer in PLR < 150 versus PLR ≥ 150 (16.8 vs 12.4 months, *P* = .012) (Table 2 and Fig. 2).

HR of patients whose PLR value was ≥ 150 over patients whose PLR value was < 150 was 1.64 (95%CI: 1.11–2.42) (*P* = .013) (Table 3).

Cox-regression analysis revealed that, when adjusted for other independent predictors of survival (age, tumor stage, operability, WBC count, neutrophil count, basophil count) (Supplementary table 2, http://links.lww.com/MD/J215), PLR still remains a significant predictor of poor survival (HR: 1.004, 95%CI: 1.001–1.006, *P* = .008).

## 4. Discussion

Our findings support the data from several meta-analysis studies on the negative prognostic role of both NLR^[19,20]^ and PLR^[21–23]^ in lung cancer patients. The prognostic significance of NLR and PLR was also reported to remain after adjusting for other prognostic factors such as gender, histology, stage, tobacco use, eastern cooperative oncology group performance status and chemotherapy approach in NSCLC patients with locally advanced disease^[24]^ and in metastatic NSCLC patients.^[25]^ In another study, elevated pretreatment NLR (>5) and PLR (>200) were reported to be associated with inferior treatment outcomes and shorter overall survival, while authors emphasized the potential utility of alternative therapeutic strategies in this subgroup of patients with elevated pretreatment levels of NLR and PLR.^[26]^ Likewise, in a study with stage III NSCLC patients treated with concurrent radiotherapy, authors reported that elevated pretreatment PLR (≥155) was associated with poorer overall survival and progression-free survival and indicated their potential benefit in identification of candidates for consolidation chemotherapy following concurrent chemo radio therapy, while they found no prognostic role of elevated NLR (≥3.2).^[5]^

In another study performed on stage IV NSCLC patients, 5 unit increase in baseline NLR and PLR was reported to be associated with an 11% and 0.5% increase in the hazard of death respectively, while NLR < 5 versus NLR ≥ 5 (13.37 vs 6.77 months for the first line treatment and 13.67 vs 5.23 months for the second-line treatment) and PLR < 185 versus PLR ≥ 185 (12.47 vs 8.77 months for the first-line treatment and 12.2 vs 10.3 months for the second-line treatment) were also associated with longer duration of survival.^[27]^

While our study included NSCLC patients of all stages in the literature there are studies that only included specific groups of NSCLC. A recent meta-analysis that examined 21 studies has concluded that preoperative elevated NLR and PLR levels may act as prognostic markers in only early stage NSCLC patients that were operated while our study has the same conclusion in all stages.^[28]^ Likewise another study that has investigated again only the operated NSCLC patients also concluded that both NLR and PLR are related to survival but PLR demonstrates a greater prognostic value than NLR.^[29]^ An interesting study which investigated newly diagnosed stage IV NSCLC patients a relationship was found between depressive symptoms and NLR,PLR increase. This study also concluded that these systemic inflammation biomarkers were prognostic for 2-year overall survival.^[30]^ A recent meta-analysis that examined 12 studies about NSCLC patients treated with immunotherapy also found evidence regarding both NLR and PLR are related to prognosis.^[31]^

Nonetheless, while the utility of NLR and PLR has been confirmed as valuable predictive parameters for tumor stages in other solid tumors such as colorectal cancer and thyroid cancer,^[32,33]^ the association of NLR and PLR with clinicopathological factors (i.e., TNM stages) has not been fully elucidated.^5^ In the current study, stage IV and IIB disease as well as inoperability were associated with poorer survival, while gender, NSCLC subtype and tumor volume had no significant impact on survival. In addition, no significant difference was noted between NLR quartiles and PLR quartiles in terms of NSCLC histological subtype or tumor volume, while advanced disease stage was the only clinicopathological factor that significantly associated both with NLR and PLR, and lower chance of operability was also associated with elevated NLR.

Our findings indicate significant prognostic role of both NLR and PLR in early-stage and locally advanced disease in predicting survival outcome, while also emphasize the likelihood of discrepancy in the prognostic yield of 2 markers in different TNM stages of NSCLC,^[5]^ such as higher prognostic yield of PLR in stage IIB disease whereas that of NLR in both stage IIB and stage IV disease.

In the current study, while neutrophilia and leukocytosis were both associated with poor survival in the univariate analysis, after multivariate analysis only leukocytosis remained as a negative prognostic factor, but neutrophilia was associated with better survival. Notably, while neutrophilia and leukocytosis were reported to be correlated with poor survival of cancer patients and several studies indicated the negative role of neutrophils during tumor progression, positive role of neutrophils in carcinogenesis has also been reported.^[5]^

Indeed, some studies have suggested combined use of NLR with other inflammatory markers, such as C-reactive protein levels to be useful in predicting the prognosis in NSCLC patients^[34]^ and superiority of NLR over PLR in predicting overall survival in NSCLC patients has been reported in other studies.^[17]^ The linear association between PLR and NLR values with significant increase in PLR from the first to fourth quartiles of NLR and significant increase in NLR from the first to fourth quartiles of PLR may indicate the likelihood of using the NLR and PLR as a combined model to better distinguish NSCLC patients with poorer survival. However, the exact prognostic role of the combined NLR and PLR analysis remains unclarified and controversial in the setting of NSCLC and needs to be tested in future studies.^[16]^

This research, however, is subject to several limitations. The first is the restrospective cohort design of the study. Because of this design our sample group consists of heterogeneous patients. We have unequal subgroups of early stage and inoperable patients that did not have the same treatment. The second limitation is related to the statistical analysis of the patients. It was hard to avoid getting biased results due to cox regression analysis we have done because we had several variables having correlation to each other. But we have done adjusted analysis for other independent predictors of survival like age, tumor stage, pathological type, operability, etc and seen that NLR and PLR still remained significant predictors of prognosis. Another limitation is the determination of the cutoff points for PLR and NLR. There is an uncertainty for these values in the literature.^[31]^ Hence determining the cutoff points is not a primary or secondary aim of this study we have determined values that were close to our median values and similar to the values of the literature and obtained balanced groups.^[35–39]^ Moreover we have used PLR and NLR values as numeric variables in Cox regression analysis and that diminished the importance of cutoff values. The findings of this study have to be seen in light of these limitations.

## 5. Conclusions

In conclusion, our findings indicate that: elevated pretreatment NLR and PLR are associated with advanced disease and poor survival in NSCLC patients. NLR and PLR values are correlated with each other. Our findings emphasize the patient age, disease stage and operability status as the potential cofactors playing a role in adverse survival impact of both inflammatory markers. Representing, simple, inexpensive and readily available inflammation-based prognostic markers, both NLR and PLR seems to have a clinical use in the early stage and locally advanced NSCLC patients in enabling early stratification of patients for treatment or optimal timing for potential treatment changes. Further studies addressing the combined models of inflammation-based prognostic factors seems to have the potential to enable accurate risk stratification and thus implementation of personalized therapy for NSCLC patients, by predicting the likely survival and treatment response outcomes.

## Author contributions

**Conceptualization:** Birsen Pinar Yildiz.

**Data curation:** Mesut Bayraktaroglu.

**Formal analysis:** Mesut Bayraktaroglu.

**Investigation:** Mesut Bayraktaroglu.

**Methodology:** Birsen Pinar Yildiz.

**Project administration:** Birsen Pinar Yildiz.

**Resources:** Mesut Bayraktaroglu.

**Supervision:** Birsen Pinar Yildiz.

**Writing – original draft:** Birsen Pinar Yildiz.

## Supplementary Material
