# Supplementary material for: Prognostic significance of neutrophil-to-lymphocyte ratio and platelet-to-lymphocyte ratio in non-small cell lung cancer
Source: Medicine (Baltimore). 2023 Jun 30;102(26):e34180. doi: 10.1097/MD.0000000000034180 (PMC10313305; doi:10.1097/MD.0000000000034180)
Supplement: Supplementary file 1 [file medi-102-e34180-s001.pdf]

Supplementary Table 1: COX REGRESSION ANALYSIS FOR PLR (MULTI VARIABLE ASSESSMENT)

In addition to the univariate survival analyzes associated with NLR, the correlation of NLR with survival was also evaluated with the multivariate Cox regression model including the variables that may affect the association of NLR with survival and adjusted effects of these variables.

	**HR**	**%95AGS**	**%95ÜGS**	***p***	**HR**	**%95AGS**	**%95ÜGS**	***p***
**NLR**	1.267	1.039	1.545	*0.019*	1.232	1.081	1.403	*0.002*
**Yaş**	1.046	1.016	1.077	*0.002*	1.043	1.017	1.070	*0.001*
**Evre**				*<0.001*				*0.000*
**1A *vs* 1B**	3.898	0.796	19.103	*0.093*	3.180	0.756	13.378	*0.115*
**1B *vs* 2A**	0.684	0.149	3.131	*0.624*	0.810	0.213	3.084	*0.757*
**2A *vs* 2B**	0.172	0.051	0.583	*0.005*	0.169	0.053	0.542	*0.003*
**2B *vs* 3A**	2.823	0.874	9.117	*0.083*	3.366	1.152	9.833	*0.026*
**3A *vs* 3B**	0.670	0.252	1.781	*0.422*	0.634	0.277	1.452	*0.281*
**3B *vs* 4**	0.433	0.211	0.889	*0.023*	0.478	0.253	0.904	*0.023*
**Tümör boyutu**	0.998	0.988	1.008	*0.663*				
**Tümör tipi**				*0.054*				*0.052*
**Adenokanser *vs* Skuamöz kanser**	1.116	0.578	2.156	*0.743*	0.909	0.515	1.606	*0.743*
**Adenokanser *vs* KHDAK**	2.180	1.060	4.483	*0.034*	1.750	0.950	3.225	*0.073*
**Operabilite**	0.188	0.076	0.468	*<0.001*	0.207	0.090	0.477	*0.000*
**WBC**	1.492	0.385	5.784	*0.563*	1.495	1.139	1.963	*0.004*
**RBC**	0.071	0.002	2.867	*0.161*				
**Hemoglobin**	1.913	0.445	8.228	*0.384*				
**Hematokrit**	1.074	0.832	1.387	*0.583*				
**Platelet**	0.999	0.994	1.004	*0.676*				
**Platokrit**	46.750	0.115	19046.435	*0.210*	13.378	0.779	229.759	*0.074*
**MCH**	0.673	0.363	1.251	*0.211*				
**Lenfosit sayısı**	1.346	0.163	11.123	*0.782*				
**Monosit sayısı**	0.604	0.196	1.860	*0.379*				
**Nötrofil sayısı**	0.557	0.134	2.309	*0.420*	0.583	0.405	0.839	*0.004*
**Lenfosit yüzdesi**	0.974	0.791	1.199	*0.802*				
**Nötrofil yüzdesi**	0.987	0.839	1.161	*0.874*				
**Eozinofil yüzdesi**	0.949	0.837	1.077	*0.420*				
