# Supplementary material for: Prognostic significance of neutrophil-to-lymphocyte ratio and platelet-to-lymphocyte ratio in non-small cell lung cancer
Source: Medicine (Baltimore). 2023 Jun 30;102(26):e34180. doi: 10.1097/MD.0000000000034180 (PMC10313305; doi:10.1097/MD.0000000000034180)
Supplement: Supplementary file 2 [file medi-102-e34180-s002.pdf]

Supplementary table 2: COX REGRESSION ANALYSIS FOR PLR (MULTI VARIABLE ASSESSMENT)

In addition to the univariate survival analyzes associated with PLR, the correlation of PLR with survival was also evaluated with the multivariate Cox regression model including the variables that may affect the association of PLR with survival and adjusted effects of these variables.

	**HR**	**%95AGS**	**%95ÜGS**	***p***	**HR**	**%95AGS**	**%95ÜGS**	***p***
**PLR**	1.008	1.002	1.014	*0.008*	1.004	1.001	1.006	*0.008*
**Yaş**	1.051	1.021	1.082	*0.001*	1.046	1.021	1.071	*0.000*
**Evre**				*<0.001*				*0.000*
**1A *vs* 1B**	3.382	0.708	16.156	*0.127*	2.228	0.547	9.081	*0.264*
**1B *vs* 2A**	0.823	0.188	3.611	*0.797*	0.905	0.238	3.434	*0.883*
**2A *vs* 2B**	0.120	0.037	0.389	*<0.001*	0.141	0.045	0.439	*0.001*
**2B *vs* 3A**	4.008	1.366	11.763	*0.011*	4.361	1.529	12.444	*0.006*
**3A *vs* 3B**	0.487	0.192	1.238	*0.131*	0.461	0.205	1.038	*0.061*
**3B *vs* 4**	0.699	0.360	1.359	*0.291*	0.783	0.434	1.414	*0.417*
**Tümör boyutu**	0.999	0.990	1.009	*0.850*				
**Operabilite**	0.224	0.093	0.535	*0.001*	0.256	0.116	0.563	*0.001*
**WBC**	2.513	0.703	8.982	*0.156*	1.389	1.069	1.805	*0.014*
**RBC**	0.154	0.003	7.590	*0.346*				
**Hemoglobin**	5.546	0.217	141.444	*0.300*				
**Hematokrit**	0.699	0.208	2.352	*0.563*				
**Platelet**	0.998	0.989	1.006	*0.618*				
**Platokrit**	1.416	0.000	19408.573	*0.943*				
**MCV**	1.131	0.638	2.005	*0.674*				
**MCH**	0.512	0.112	2.336	*0.388*				
**MPV**	1.257	0.828	1.907	*0.283*				
**Lenfosit sayısı**	0.834	0.126	5.498	*0.850*				
**Nötrofil sayısı**	0.355	0.091	1.380	*0.135*	0.709	0.524	0.958	*0.025*
**Bazofil sayısı**	5.347	1.231	23.219	*0.025*	5.473	1.370	21.855	*0.016*
**Lenfosit yüzdesi**	1.076	0.876	1.320	*0.487*				
**Nötrofil yüzdesi**	1.076	0.923	1.253	*0.349*				
